# Aminolysis of Poly-3-Hydroxybutyrate in N,N-Dimethylformamide and 1,4-Dioxane and Formation of Functionalized Oligomers

**DOI:** 10.3390/polym14245481

**Published:** 2022-12-14

**Authors:** Anatoly Nikolayevich Boyandin, Viktoriya Aleksandrovna Bessonova, Natalya Leonidovna Ertiletskaya, Anna Alekseevna Sukhanova, Taisiya Aleksandrovna Shalygina, Alexander Alexandrovich Kondrasenko

**Affiliations:** 1 Scientific Laboratory “Smart Materials and Structures”, Reshetnev Siberian State University of Science and Technology, 433/1 Ul. Semafornaya, Krasnoyarsk 660059, Russia; 2 Institute of Biophysics SB RAS, Federal Research Center “Krasnoyarsk Science Center of the Siberian Branch of the Russian Academy of Sciences”, 50, b. 50, Akademgorodok, Krasnoyarsk 660036, Russia; 3 School of Fundamental Biology and Biotechnology, Siberian Federal University, 79 Svobodny pr., Krasnoyarsk 660041, Russia; 4 Institute of Chemistry and Chemical Technology SB RAS, Federal Research Center “Krasnoyarsk Science Center of the Siberian Branch of the Russian Academy of Sciences”, 50, b. 24, Akademgorodok, Krasnoyarsk 660036, Russia

**Keywords:** poly-3-hydroxybutyrate, polyesters, bifunctional amines, aminolysis, functionalized oligomers

## Abstract

The degradation pattern of bacterial poly-3-hydroxybutyrate (PHB) in dimethylformamide (DMF) and dioxane solutions at 100 °C assisted by ethylenediamine, 1,4-diaminobutane and monoaminoethanol was studied. When diamines were introduced into the PHB solution in DMF in the amount of 1 mol of the reagent to 5 or 10 mol of PHB monomers, a rapid decrease in the molecular weight of the polymer was observed. The initial value of the weight average molecular weight (M_w_) 840 kDa had decreased by 20–30 times within the first 10–20 min of the experiment, followed by its gradual decrease to several thousand Da. When a similar molar quantity of aminoethanol was added, the molecular weight decreased slower. PHB had been degrading much slower in the dioxane solution than in DMF. By varying the number of reagents, it was possible to reach stabilization of the M_w_ at 1000–3000 Da when using diamines and 8000–20,000 Da using aminoethanol. ^1^H NMR analysis of the oligomers revealed of amino and amido groups forming in their structure. From the opposite end of the polymer chain, residues of 3-hydroxybutyric, crotonic and isocrotonic acids were formed during degradation. Differential scanning calorimetry indicated that after oligomerization there was a decrease in the melting point from 178 °C to 140–170 °C depending on the decrease in the molecular weight. The method proposed can be used for obtaining aminated PHB oligomers.

## 1. Introduction

Obtaining polymers bearing active functional groups in their structures is a significant trend in macromolecular chemistry. Such functionality can be used for chemical embedding of non-polymer molecules such as drugs, vitamins, cofactors and other biologically active compounds for medical applications into polymer chains [1]. Other groups include dyes [2], enzymes and other catalytic agents, factors providing electrical conductivity, photosensitivity, etc. [3]. Depending on the nature of such “embedment”, the derivatives obtained are of high importance for medicine [4], analytical chemistry, biochemical diagnostics, molecular biological procedures [5], design of markers, electro- and photochemical sensors, etc.

Polyhydroxyalkanoates (PHAs) of bacterial origin have a special place among polymers in terms of their biological characteristics [6]. Due to the gradual degradation in the body to natural for metabolic processes or, at least, non-toxic hydroxy acids, this group of polyesters is referred to as the so-called biopolymers. Such polymers exhibit high biological compatibility, primarily in relation to the internal environment of the human body and higher animals. They are possibly to be used in different fields of biomedicine, including the development of various kinds of implants and bioengineering structures designed for contact with the internal environment of the body, as well as the production of new generation drugs with targeted delivery, as well as external medical devices, such as wound healing materials. All these areas of application may include the need for biologically active compounds (BACs) of different natures, which depend on the specific purposes of application, to be introduced into polymer structures. This requires the study of strategies for the intermediate modification of PHAs to introduce functional groups that are promising for further attachment of BACs.

In recent years, an important trend in biocompatible polyesters modification has been the obtaining of their oligomers for further use in several fields [7]. In particular, such oligomers can be used in chain elongation reactions or for copolymerization with oligomers of a different nature to obtain new polymers [8,9,10] or for the addition of low molecular weight compounds [11]. Thus, for the formation of microparticles that can be used for controlled drug delivery, it is desirable to use polymers with relatively low molecular weight (around thousands of Da) [12]. When obtaining blends of biodegradable polymers, e.g., PHA-PLA blends, the inclusion of low molecular weight PHA provides better miscibility of the components, has a plasticization and lubrication effect, and facilitates the formability of these blends into the desired shape [13]. Therefore, the combination of controlled oligomerization, which allows for the obtaining of oligomers of a specific molecular weight, with the formation of active functional groups at the end (in particular, amine and hydroxyl) is important for the further binding of BACs of various natures.

In addition, the most common representatives of PHA (including poly-3-hydroxybutyrate, PHB) practically do not possess functional groups that can be used for covalent addition of other compounds [14]. The reactions involving the ester group inevitably lead to a polyester chain scission and a drop in the molecular weight of the polymer. Therefore, controlled oligomerization is essential for the concomitant functionalization of polymer molecules.

At present, several techniques have been developed to obtain such PHA oligomers. The simplest and relatively old technique is the thermal degradation of PHA at a relatively low temperature, leading to the gradual degradation of polyester molecules with the formation of unsaturated acid residues (in the case of PHB—crotonic) and carboxyl groups at the corresponding ends [15]. During further heating at high temperatures, this process results in volatile monomers, dimers and trimers [14]. Similar oligomers were obtained in the result of microwave-assisted PHB degradation [16]. For this reaction, a mechanism involving cis-elimination with the formation of an intermediate of an ester in the form of a six-membered ring has been studied in detail [17]. One of the modifications of this method includes the fusion of polyesters with salts, for example acetates [18], which causes it to be more convenient to control the depolymerization rate and can facilitate the production of oligomers with specific physical and mechanical characteristics (for example, a foamed polymer when using carbonates). The end groups of crotonic acid can be subjected to subsequent modifications by oxidation of the double bonds to obtain a wide variety of functional end groups, such as hydroxyl, carboxyl, oxirane or aldehyde groups [19]. Besides, methods for the production of oligomers with two terminal hydroxyl groups by alcoholization of high-molecular PHAs with diols and triols, such as ethylene glycol and glycerin [20], 1,3-propanediol [21] and 1,4-butanediol [21,22] (in the presence of a catalyst, for example, p-toluenesulfonic acid or dibutyltin dilaurate) have been developed. In this case, varying the conditions (primarily time) of alcoholysis causes it to be possible to obtain macrodiols (s.c. PHB-diols) with different chain sizes (and, accordingly, molecular weight). Alternatively, at present, PHB-diols are also obtained biotechnologically by the addition of diols in the fermentation broth during controlled PHB synthesis [10]. To obtain star-shaped oligomers for further copolymerization with ε-caprolactone, PHB was also subjected to alcoholysis with polyols such as trimethylol propane, pentaerythritol and dipentaerythritol [9]. The usual hydrolysis of PHA is also possible under acidic [23] and especially alkaline conditions. In this case, in the presence of strong bases, the formation of residues of both hydroxyacids and unsaturated acids occur. The formation of the latter, in contrast to thermal degradation, proceeds by the mechanism of beta-elimination [24,25]. Another method for carrying out the hydrolysis of high molecular weight PHB is application of microbial depolymerases in vitro [26]. The reduction in PHAs with sodium or lithium borohydride is also known [27,28]. This is usually carried out in order to reduce their molecular weight and for the further use of oligomers, e.g., in the production of nanoparticles [27] and other tailor-made biodegradable materials [29]. A relatively small range of functional groups obtained (carboxyl, hydroxyl, or double bonds) is a feature of the methods described above. This limits the possible range of reactions or makes it necessary to introduce additional stages that negatively affect the yield and purity of the products.

At the same time, a method for increasing the hydrophilicity and biocompatibility of the products created with various biocompatible polyesters of hydroxycarboxylic acids, including PHA, by treating their surfaces with diamines is known. These diamines are embedded into the polymer surface due to aminolysis of its ester bonds and form free amino groups on the surface. Such a treatment can increase surface hydrophilicity, improve cell adhesion, proliferation and cellular functions, dominance of stem cell differentiation and isolation of certain subgroup of cells, demonstrating the versatility of the aminolysis-based polyester surface modification in biomedical applications [30]. The demonstrated efficacy of the method described in this study allows us to consider its use for the directed obtaining of functionalized PHA oligomers.

The aim of this work was to study the degradation of poly-3-hydroxybutyrate in the form of high-temperature solutions in N,N-dimethylformamide and 1,4-dioxane assisted by amino compounds with two functional groups in their structure—the diamines ethylenediamine and 1,4-diaminobutane and the amino alcohol monoethanolamine.

## 2. Materials and Methods

### 2.1. Materials

Poly-3-hydroxybutyrate (the weight average molecular weight M_w_ 840 kDa; polydispersity 2.70) was synthesized in Siberian Federal University according to previously described technology [31]. The following reagents were used: N,N-dimethylformamide for synthesis from Roth, Germany; reagent grade 1,4-dioxane, ACS grade isopropyl alcohol and purified chloroform from ECOS-1, Russia; 1,4-diaminobutane (98+% purity) from Alfa Aesar, Kandel, Germany; ethylene-1,2-diamine for synthesis and monoethanolamine for synthesis from Merck, Darmstadt, Germany.

### 2.2. Methods

#### 2.2.1. Aminolysis and Oligomeric Samples Isolation

For aminolysis, 1.72 g (0.02 mol in recalculation on monomers, i.e., on ester bonds) of PHB was dissolved in 86 mL of dimethylformamide or dioxane at a temperature of 100 °C to obtain a 2% (*w*/*v*) solution. After the polymer was completely dissolved, ethylenediamine (EDA; molar mass 60.1 g × mol^−1^), 1,4-diaminobutane (DAB; 88.15 g × mol^−1^) or monoethanolamine (MEA; 61.08 g × mol^−1^) were added at a given concentration calculated in relation to the moles of the reagent to the number of monomers in the polymer. The molar mass of PHB monomer (–O–CH(CH_3_)–CH_2_–CO–) in the esterified state is 86 g/mol. After the reagents were added, the mixtures were exposed at 100 °C for 10 h with hourly sampling.

The oligomers were isolated by precipitation with four times the volume of isopropyl alcohol related to the volume of dimethylformamide, followed by filtration and washing with isopropyl alcohol on a glass filter.

For FTIR spectroscopy (Section 2.2.5), the products of complete PHB degradation assisted by the excess of amines were obtained. For this, 0.43 g of PHB (0.005 mol in recalculation on monomers, i.e., on ester bonds) were dissolved in 21.5 mL of dimethylformamide or dioxane. 0.01 mol of EDA, DAB or MEA were added to the obtained solution. The mixtures were heated during 3 h followed by the solvents and the excess of the amines were distilled on a rotary evaporator at 90 °C temperature and 1 mbar pressure during 3 h. The obtained residues were redissolved in methanol, the solutions were filtered and methanol was also distilled.

#### 2.2.2. Molecular Weight Analysis

The change in the molecular weight of the polymer over time was estimated, depending on the solvent and type and concentration of the reagent. The molecular weight and molar mass distribution of the PHB oligomers were analyzed by gel permeation chromatography using Agilent 1260 Infinity chromatograph (Agilent Technologies, Santa Clara, CA, USA) equipped with an isocratic pump, an autosampler and a refractometric detector. The Agilent PLgel Mixed-C column was used. Chloroform was used as an eluent and the flow rate and temperature were 1.0 mL/min and 40 °C, respectively. The sample volume was 50 µL and the polymer concentration in the solution was 5 mg/mL. The calibration curve was obtained with a set of Agilent EasiVial PS-H narrowly dispersed polystyrene standards. The weight-average molecular weight (M_w_) was estimated.

#### 2.2.3. Differential Scanning Calorimetry (DSC)

The calorimetric measurements of the samples were carried out using a differential scanning calorimeter DSC25 (TA Instruments, New Castle, DE, USA) and sealed aluminum Tzero pans. The measurements were carried out in a nitrogen atmosphere at a flow rate of 70 mL/min in the temperature range from −20 to 200 °C with a heating rate of 10 °C/min. The first heating continued up to 190 °C at a rate of 20 °C/min and then the sample was cooled down to −20 °C at a rate of 3 °C/min. The results were processed using the TRIOS v. 5.00.44608 software package (TA Instruments, New Castle, DE, USA).

#### 2.2.4. Nuclear Magnetic Resonance (NMR) Spectroscopy

The ^1^H NMR spectra were recorded at 25 °C using a Bruker Avance III 600 (Bruker, Bremen, Germany) spectrometer at 600 MHz operating frequency in Krasnoyarsk Regional Center of Research Equipment of Federal Research Center “Krasnoyarsk Science Center SB RAS”.

For the study, approximately 10 mg of a dry sample was dissolved in 1 mL of CDCl_3_ and incubated until the polymer was completely dissolved and dispersed (12 h). For ^1^H NMR and COSY spectra, a five-fold dilution of the resulting solution was used. ^1^H NMR spectra were obtained by accumulating 64 scans with a relaxation delay of 8 s. After the Fourier transform, spectra with a sweep of 24 ppm were obtained. For recording double-quantum filtered COSY spectra, 128 experiments with 8 scans each were performed with a delay of 5 s.

#### 2.2.5. FTIR (Fourier Transformed Infra-Red) Spectroscopy

The chemical structure of the samples was studied using infrared spectroscopy with a Fourier transform using the Nicolet iS10 FTIR spectrometer (Thermo Scientific, Waltham, MA, USA) and the ITX Smart prefix (Thermo Scientific, USA) with a diamond crystal by a disturbed total internal reflection method (DTIR). The analyses were carried out with a spectral resolution of 4 cm^−1^, averaged over 32 scans, in the range of 4000–400 cm^−1^. The obtained IR-Fourier spectra were processed in the OMNIC software applying an advanced correction of disturbed total internal reflection.

## 3. Results

### 3.1. Change in the Molecular Weight of PHB during Amine-Assisted Degradation

In the preliminary experiments, the evaluation of PHB solubility in DMF and dioxane was performed since no relevant quantitative data could be found in the literature except the very fact of this possibility. PHB was introduced in the corresponding solvents in a 2% ratio (*m*/*v*) and the resulting blends were heated during 1 hr at the given temperature starting from 50 °C in increments of 10 °C. It was found that PHB swelling in both solvents occurs at 60 °C, when dissolution occurs at 100 °C and 90 °C in DMFA and dioxane, respectively. Taking into account the boiling point of 101 °C of dioxane at normal pressure, the temperature of 100 °C was chosen for the further experiments.

Initially, an experiment was conducted to assess the rate of aminolysis. EDA or DAB were added in equimolar amounts with respect to the number of PHB monomers to a solution of PHB in dimethylformamide. As a result, quantitative degradation of the polymer was observed for the short time (within one hour). It was impossible to isolate oligomers under these conditions. When dioxane was used as a solvent and the same ratios of reagents were maintained, the precipitated products of degradation by EDA or DAB were released in insignificant amounts within 6–7 h. When using MEA, the release of degradation products was observed for both solvents up to the end of the experiment.

**Figure 1:**
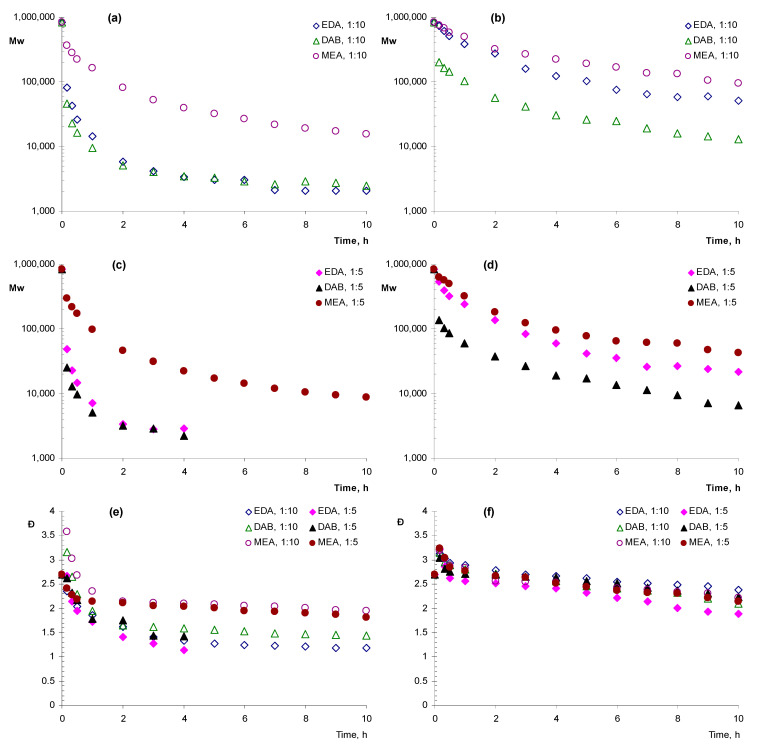
Dynamics of the weight average molecular weight (**a**–**d**) and polydispersity (**e**,**f**) during aminolysis of poly-3-hydroxybutyrate (PHB) in dimethylformamide (**a**,**c**,**e**) and dioxane (**b**,**d**,**f**) with the addition of ethylenediamine (EDA), 1,4-diaminobutane (DAB) and monoethanolamine (MEA) in the different ratios amine:monomer.

In the further experiments, lower concentrations of amines were used, with amine:monomer ratio equal to 1:5 and 1:10. The analysis of changes in the molecular weight of the polymer affected by amines in a dimethylformamide solution demonstrated that the most rapid decrease in molecular weight was caused by the addition of DAB or EDA, compared with MEA. In all the cases of aminolysis, a rapid drop in molecular weight in the first 1–2 h of the experiment and further stabilization of the achieved values at a relatively constant level until the end of the experiment were observed. At the same time, the values of the molecular weight at which stabilization occurred differed depending on the conditions of aminolysis. With a 1:10 ratio of the number of diamines to ester bonds (Figure 1a), after 10 min, a decrease in M_w_ down to 81.9 kDa and 45.6 kDa was observed for EDA and DAB, respectively (Table 1). One hour later, the corresponding values were 14.3 kDa and 9.5 kDa and, at the end of the experiment, they reached 2.1 kDa and 2.5 kDa. When MEA was added, slower degradation was observed. Specifically, M_w_ decreased to 362 kDa and 161 kDa in 10 min and in an hour, respectively, and its final value was 15.6 kDa.

With an increase in the molar amino:polymer ratio up to 1:5 (Figure 1c), an increase in the rate of polymer degradation was observed. After the fourth hour of the experiment, oligomeric products were not released during aminolysis with EDA and DAB and the final products at the time of isolation had the values of M_w_ 2.9 and 2.2 kDa, respectively. When MEA was added, oligomeric products precipitated until the end of the experiment (10 h). The value of M_w_ was 297 kDa, 96.3 kDa h and 8.8 kDa after 10 min, an hour and at the end of the experiment, respectively.

The use of dioxane as a solvent led to a significant decrease in the degradation rate compared with solution in dimethylformamide. Under these conditions, DAB appeared to be the most active as, one hour after its addition, the M_w_ of dissolved PHB decreased to 103 kDa and 58.9 kDa for the ratios of reagent to polymer 1:10 (Figure 1b) and 1:5 (Figure 1d), respectively (Table 1). In the case of the addition of 1,2-ethylene diamine, the similar values were 388 kDa and 243 kDa. Comparable values of M_w_ 498 and 322 kDa were observed after an hour when MEA was added. This trend continued until the end of the experiment. In the presence of DAB, on the tenth hour of the experiment M_w_ of oligomeric products were 13.0 kDa and 6.7 kDa at amine:monomer ratios 1:10 and 1:5, respectively. In the case of EDA, the respective values were 50.6 and 21.9 kDa and, in the case of MEA they were 95.6 and 42.6 kDa, respectively.

It is worth noting that, during the experiment, the polydispersity of all the samples was decreasing and this decrease generally correlated with the decrease in molecular weight. The largest decrease in Ð from 2.70 in the beginning of the experiment to 1.14–1.42 in its end was observed for in DMF in the presence of the diamines (Figure 1e). In the same solvent in the presence of MEA after 10 h Ð was at the level of 1.81–1.94. In dioxane (Figure 1f), the decrease in Ð was generally less dramatic and comparable for different samples with ultimate values at 1.82–2.39.

### 3.2. Characteristics of Oligomeric PHB Samples

The samples isolated after 10 h treatment with amines at an amine:monomer ratio 1:5 were chosen for further research. The EDA/DMF and DAB/DMF samples, the release of which at the amine:monomer ratio 1:5 did not occur after 10 h, were obtained by additional 1.5 h exposure under the same conditions. Their M_w_ values were 5.2 and 3.9 kDa, respectively.

**Figure 2:**
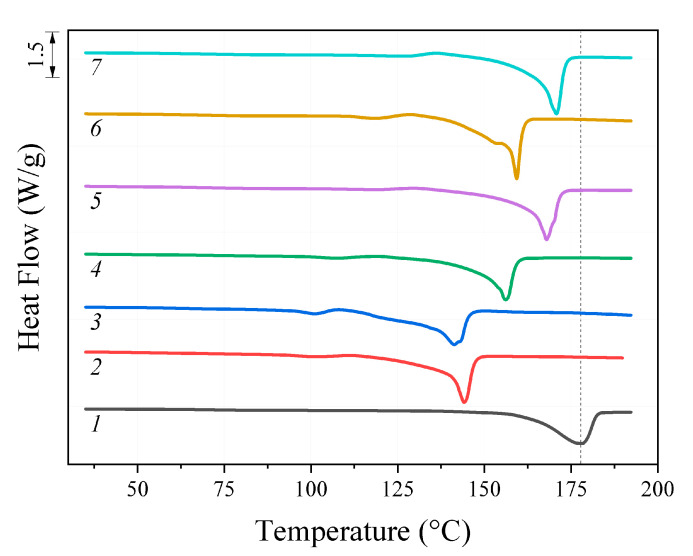
DSC thermograms: 1—initial PHB; oligomers after treatment: 2—EDA/DMF; 3—DAB/DMF; 4—MEA/DMF; 5—EDA/dioxane; 6—DAB/dioxane; 7—MEA/dioxane.

The results of the melting point (T_m_) analysis of the initial PHB and its oligomers, determined using the DSC method, are shown in Figure 2. For all the oligomers studied, the obtained Tm values were lower than for the initial polymer and correlated with the molecular weight. Thus, the lowest T_m_ value amounting to 141 °C was observed for DAB/DMF sample with M_w_ 3.9 kDa. For EDA/DMF sample close to it in M_w_ (5.2 kDa), T_m_ was 143 °C. With the increase in M_w_ values, the T_m_ values also increased. The highest melting point value of 178 °C was measured for the initial high-molecular polymer (840 kDa).

**Figure 3:**
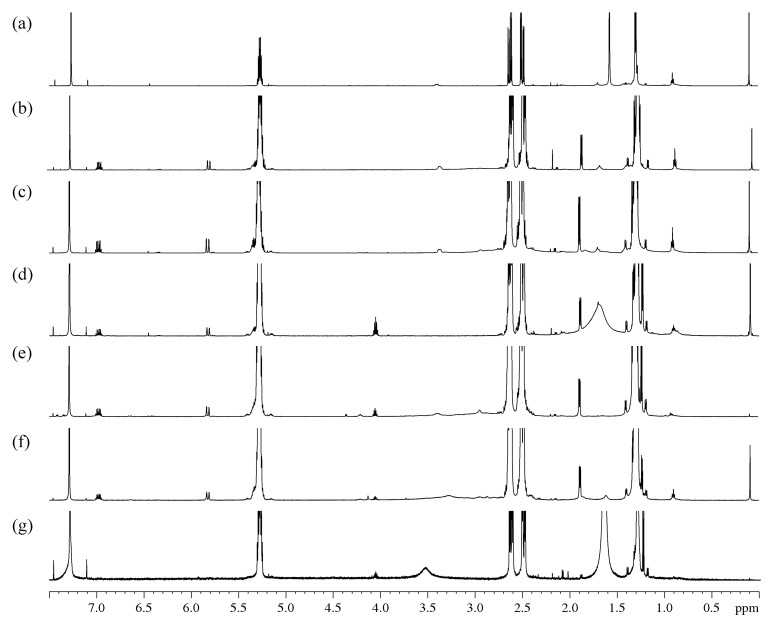
Fragments ^1^H NMR spectra: (**a**)—initial PHB; oligomers after treatment: (**b**)—EDA/DMF; (**c**)—DAB/DMF; (**d**)—MEA/DMF; (**e**)—EDA/dioxane; (**f**)—DAB/dioxane; (**g**)—MEA/dioxane.

The ^1^H NMR spectra (Figure 3 and Figure S1) and COSY correlation spectra (Figure S2) were obtained for the initial PHB and its oligomers isolated after its degradation by amines.

On the ^1^H NMR spectra of the control sample (Figure 3a), the main peaks are represented by PHB with known spectral characteristics (δ_Me_ 1.28, δ_CH2a_ 2.48, δ_CH_ 5.27, δ_CH2b_ 2.61, J_CHMe_ 6.4, J_CHCH2a_ 5.9, Jgem_CH2_ 15.7, J_CHCH2b_ 7.3). The spectra also revealed an insignificant number of monomeric units of 4-hydroxybutyrate (δ_αCH2_ 1.70, δ_βCH2_ 1.42, δ_γCH2_ 3.40), 3-hydroxyvalerate (δ_CH3_ 0.85) and hydroxy acids with a chain length of six or more (δ_CH3_ 0.89).

A series of multiplets was found on the ^1^H spectra of samples obtained by the degradation of PHB at 6.96 ppm (doublet of quartets, 3 J = 15.6, 3 J = 6.9 Hz, 1H), 5.80 ppm (doublet of quartets, 3 J = 15.6, 4 J = 1.6 Hz, 1H) and 1.87 ppm (doublet of doublets, 3 J = 6.9, 4 J = 1.6 Hz, 3H). In addition, the multiplets similar in chemical shifts and splitting patterns to the fragment described above were found, which were attributed to its isomeric form: 6.33 ppm (doublet of quartets, 3 J = 11.1, 3 J = 7.3 Hz, 1H), 5.75 ppm (doublet of quartets, 3 J = 11.4, 4 J = 1.6 Hz, 1H) and 2.13 ppm (doublet doublets, 3 J = 7.2, 4 J = 1.7 Hz, 3H). The binding of these protons into a single spin systems is confirmed by the ^1^H-^1^H COSY spectrum. Using the spectra of heteronuclear correlations of ^1^H-^13^C, some chemical shifts belonging to the fragment were found: 17.95; 122.70; 144.82; ~166 ppm. Based on these data, the main and isomeric form of the considered fragment was attributed to the residues of crotonic and isocrotonic acids.

The obtained oligomer spectra peaks in the area of 3.9–4.5 ppm were also detected. All of them are multiplets with an estimated number of spin-spin interaction (SSI) constants of more than three (δ_1_ = 4.03; δ_2_ = 4.19; δ_3_ = 4.34 ppm). In addition, there are also several peaks in the area of 6.2–6.6 ppm beyond the one previously attributed to isocrotonic acid. The analysis of the COSY spectrum showed that the doublet of triplets at 6.38 ppm is associated with SSI of doublet of doublets with a chemical shift at 4.34 ppm and a doublet at 6.63 ppm. Taking into account the expected chemical transformations and the specificity of the chemical shift area for the fragments found earlier, these multiplets were attributed to the amide of 3-hydroxybutyric acid. The doublet of doublets at 4.34 ppm was attributed to the amide proton. On the COSY spectrum, the multiplet at 4.03 ppm shows the presence of an SSI with an area of methyl shifts of PHB. Based on these correlations, these multiplets were also attributed to amide protons. The resonance at 4.19 ppm is assigned to the –CH– from the hydroxyl end of 3-hydroxybutyric acid.

Obviously, when an amide is formed, the spectrum must include peaks corresponding to the alkyl chain of the amine used in the modification. In spectra of oligomers obtained by treatment with EDA и DAB, an expanded peak at 2.92 ppm could be observed, which corresponds to the α-methylene group of alkylamines. In addition, in the COSY spectrum of the DAB derivatives (Figure 3c,f), there is a correlation of this peak with a previously unrelated peak at 1.60 ppm, which may correspond to β-methylene protons of DAB. The presence and integral intensity of amide protons in the range of 3.9–4.5 ppm can be considered reliable analytical characteristics. Due to the smaller overlap, the degree of aminolysis of PHB can be estimated by the methyl group doublet at 1.21 ppm, which, based on the COSY data, is attributed to the amide fragment.

A characteristic feature of the ^1^H NMR spectra of the samples after the degradation assisted by MEA (Figure 3d,g) is the presence of a single multiplet in the area of previously found amide protons at 4.03 ppm. Using the attributions created for EDA and DAB derivatives, it can be argued that this peak refers to fragments with hydroxyalkanoate amides with asymmetric substitution of the N-alkyl chain. The spectrum includes a doublet of the methyl group of amides at 1.21 ppm and multiplets of crotonic and isocrotonic acid. The wide peak at 1.5–1.8 ppm was attributed to the methylene groups of MEA with the possible superposition of free water protons.

The PHB degradation in dioxane and DMF occurs with the formation of a smaller fraction of crotonic acid compared to terminal monomer units of 3-hydroxybutyric acid. The proportion of these monomer units depends on the type of amine and accounts for 2:3 for EDA and 1:2 for DAB. The fraction of amide units was determined based on the nearest to CH2-groups amines. It can be noted that the proportion of PHB terminal groups and –NH–CH2– groups in the case of EDA almost not affected by the solvent used (1:1 in DMF and 3:2 in dioxane). For DAB, the corresponding ratios were 1:1 in DMF and 1:3 in dioxane.

The assignments of ^1^H chemical shifts of the PHB groups are listed in Table 2. The performed calculations of correlation between end groups showed that the number of terminal dehydrated units of crotonic and isocrotonic acid to the number of terminal groups of the hydroxybutyric acid ratio is approximately 1:1. The change in crotonic and isocrotonic acids units ratio depended on both solvent and used amine. If in DMF during aminolysis with EDA this ratio was equal to that known for thermal degradation (10:1), then in dioxane it was approximately 15:1. In aminolysis with diaminobutane, this ratio was 20:1 regardless of the solvent.

Based on this, it can be argued that aminolysis of the PHB polymer chain occurs with the formation of an acid amide and hydroxybutyric acid. The competing process of chain degradation is thermolysis with dehydration of PHB to crotonic acid.

**Figure 4:**
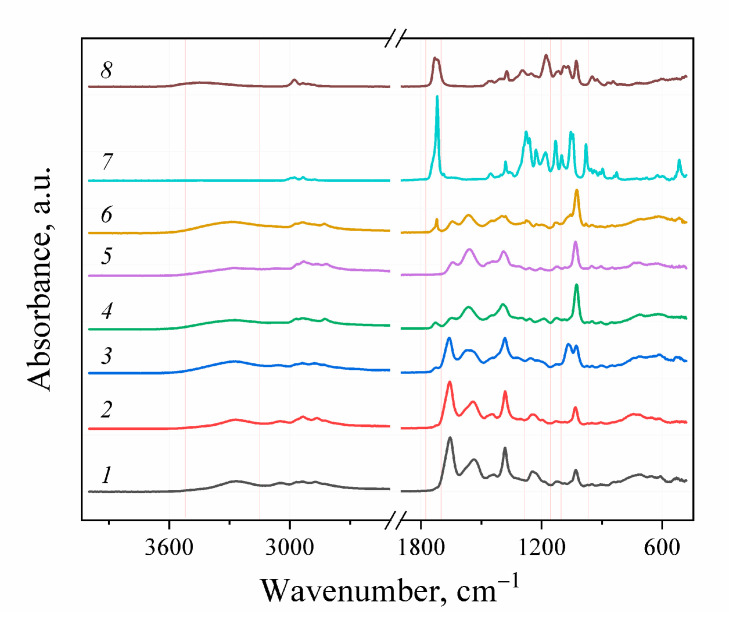
FT-IR spectra: 1–6—products of full PHB depolymerization with excess of amines: 1—EDA/DMF; 2—DAB/DMF; 3—MEA/DMF; 4—EDA/dioxane; 5—DAB/dioxane; 6—MEA/dioxane. 7—initial PHB; 8—ethyl-3-hydroxybutyrate.

**Figure 5:**
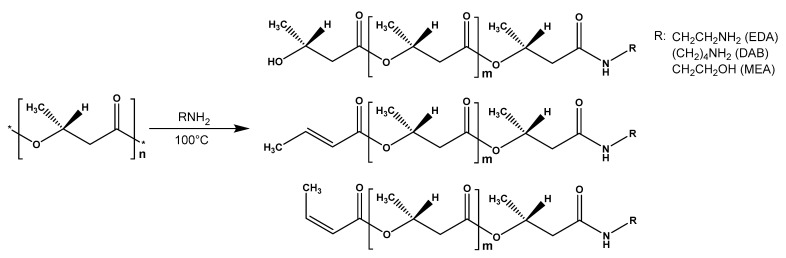
Scheme of PHB aminolysis (according to NMR data).

The FTIR-spectroscopy did not allow for the positive identification of the difference between the obtained experimental samples and the control due to the low sensitivity of the spectrometer used. For FTIR detection of the functional groups forming during aminolysis, the complete PHB degradation assisted by the excess of amines and solvents and reagents distillation followed by the separation of the obtained aminated PHB monomers were performed (Figure 4). The initial PHB and ethyl-3-hydroxybutyrate stretch vibrations of C=O groups characteristic for alcohol esters could be observed at 1719–1725 cm^−1^. For the aminated samples, the corresponding absorption band was not observed. Instead, stretching vibrations in the area of 3268–3290, 1640–1659 and 1541–1564 cm–1, which can be correlated with stretching vibrations of νN–H, νC=O and bending vibrations δN–H secondary amide groups, respectively. For the samples, obtained in reaction with diamines, the appearance of the peak in the area of 1083–1086 cm^−1^ can be observed, which is characteristic for the primary amino group. In contrast, for the samples obtained in reaction with MEA stretching vibrations at 1056–1067 cm^−1^ are observed. Probably, these vibrations related to the primary alcohol group of the aminoethanol fragment in obtained amides. Thus, the aminolysis of PHB under these conditions occurs according to the scheme shown in Figure 5.

## 4. Discussion

In the present study, the mechanism of PHB degradation assisted by bifunctional amines and the possibility of its use to obtain oligomers with aminoalkyl or hydroxyalkyl groups at the “carboxyl” end was investigated. The aminolysis of esters assisted by amines is one of the conventional methods for the production of amides [32]. When esters interact with amides, the ester bond breaks and amide is formed. The alcohol part of the ester is usually released as a free alcohol. In the case of polyesters, this reaction involves the fragmentation of the polymer chain and the formation of amides containing N-substituents corresponding to the amines used in the reaction. Although the reaction itself is quite slow under normal conditions, breaking even a small number of ester bonds should be enough to produce oligomers with a molecular weight of several thousand Da.

**Figure 6:**
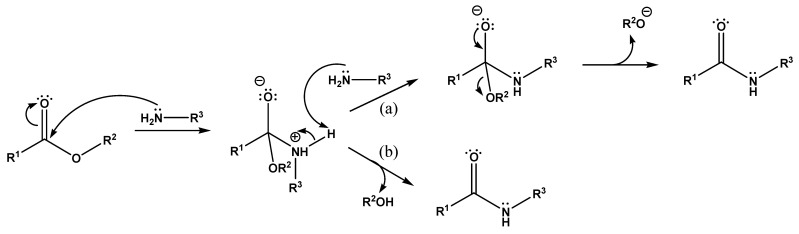
Mechanism of ester aminolysis: (**a**) through an intermediate complex with a second amine molecule, and (**b**) due to the decay of the initial intermediate.

The mechanism of ester aminolysis was previously proposed and developed in several studies [33,34,35,36]. According to the proposed scheme (Figure 6), the aminolysis reaction proceeds via two different competing mechanisms: through an intermediate complex with a second amine molecule (second-order reaction, Figure 6a) and without its formation, due to the decay of the initial intermediate (first-order reaction, Figure 6b), and the probability of the reaction proceeding along the first path is generally higher. In fact, the second amine molecule plays the role of a catalyst.

**Figure 7:**
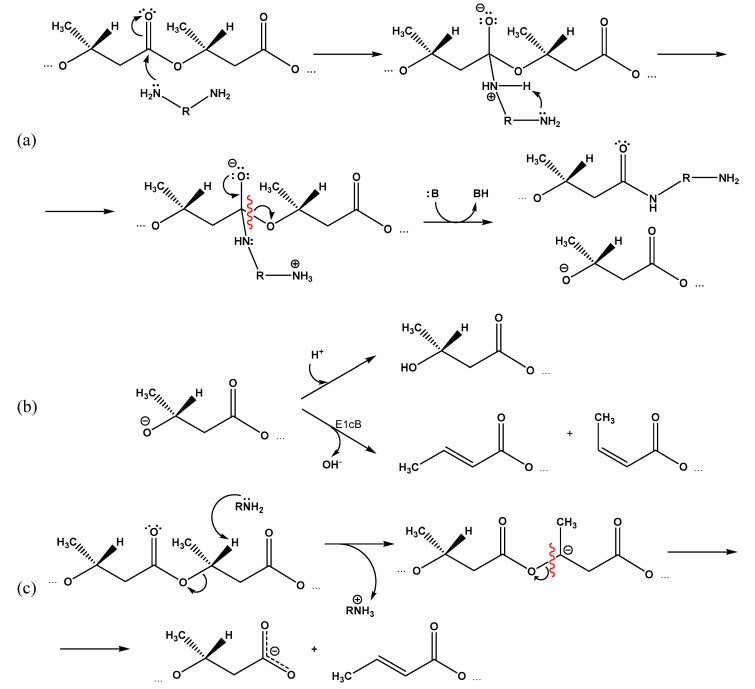
Proposed mechanisms of PHB aminolysis by diamines: (**a**) formation of an intermediate complex involving the second amino group of the diamine and its degradation; (**b**) transition of intermediate alcoholate anion in stable state due to adjoining of proton or elimination of hydroxide anion (B—any appropriate proton acceptor); (**c**) possible competitive mechanism for polymer chain degradation assisted by amines.

It can be assumed that in the case of EDA and DAB, the second amino group of the reagent is able to participate in the formation of the indicated transition state (Figure 7a), which facilitates the reaction compared to ethanolamine, especially considering the relatively low concentrations of amines in the medium.

At the same time, the esters of either phenols [33,34,35] or primary alcohols [36] were studied in the above-mentioned studies. Taking into account the greater disposition of secondary alcohols to elimination reactions compared with primary ones, under the reaction conditions under study, there is a high probability of degradation of the intermediate secondary alcoholate anion according to the E1cB elimination mechanism with the release of the hydroxyl anion, double bond closure, and the formation of a 2-butene (crotonic) and cis-2-butene (isocrotonic) acids residues (Figure 7b).

The decreased calculated content of amino groups compared to the total content of terminal residues of crotonic and 3-hydroxybutyric acid suggests a third mechanism of PHB cleavage assisted by amines, which occurs by the E2 mechanism and is associated with the elimination of a proton from the 3-hydroxybutyric acid monomer in the β-position. The resulting intermediate carbanion breaks with the scission of the polymer chain and forms a free (dissociated) carboxyl group at one end and a crotonic acid residue at the other (Figure 7c).

The obtained patterns in PHB aminolysis and in the obtaining of its oligomers show the dependence of the reaction rate on the concentration of the aminating reagent, as well as on its type and the solvent used. At the same temperature (100 °C), the highest reaction rate was shown when DAB was used as a reagent and DMF as a solvent. The least active reagent was MEA. At the same time, if in the DMF medium the dynamics of degradation with the addition of EDA and DAB practically coincided, then in dioxane medium the activity of EDA was almost similar to that of MEA. When adding EDA, DAB and MEA in the 1:10, 1:10 and 1:5 amine:monomer ratios, respectively (which, taking into account the two times lower number of amino groups in the MEA, allows us to assert the same concentration of amino groups), the final values of the weight-average molecular weight in dioxane were 50.6, 13.0 and 42.6 kDa, respectively, whereas for the DMF medium similar values were 2.1, 2.5 and 8.8 kDa. It can be expected that by varying the concentration and exposure time, oligomers can be obtained in a wide range of molecular weights in various variants of the reagent/solvent combination.

The decrease in molecular weight was followed by a decrease in polydispersity, which in general is characteristic for the degradation of high molecular weight polymers in solution [37]. However, at the beginning of the experiment, a slight transient increase in polydispersity up to 3.6 was mainly observed (Figure 1e,f). We assume that this is due to nonuniform degradation of the polymer at the time of adding the reagent and up to the complete mixing of the components.

## 5. Conclusions

The study of patterns of PHB degradation assisted by bifunctional amines showed a decrease in the activity of amines in the row 1,4-diaminobutane > ethylenediamine > aminoethanol. A slower rate of the process in the dioxane medium compared with DMF was also shown. The chain scission occurs with the formation of a double bond (in the form of crotonic or isocrotonic acid) at the “alcohol” end and the addition of amine to the “carboxyl” end of the oligomeric chain followed by formation of an amide bond. The studied reaction can be used for obtaining aminated PHB oligomers.

## Figures and Tables

**Table 1:** Dynamics of the weight average molecular weight (M_w_, kDa) during aminolysis depending on solvent, amine and its molar ratio to PHB monomers. Initial M_w_ was 840 kDa.

Solvent	Time	Reagent and Molar Ratio					
		EDA		DAB		MEA	
		1:10	1:5	1:10	1:5	1:10	1:5
DMF ^1^	10 min	81.9	48.9	45.6	25.4	362	297
DMF	1 h	14.3	7.06	9.49	5.04	161	96.3
DMF	4 h	3.37	2.88	3.48	2.25	39.2	22.2
DMF	10 h	2.07	–	2.47	–	15.6	8.80
Dioxane	10 min	731	534	199	138	754	632
Dioxane	1 h	388	243	103	58.9	498	322
Dioxane	4 h	124	60.3	30.72	19.3	220	94.4
Dioxane	10 h	50.6	21.9	12.97	6.69	95.6	42.6

^1^ DMF—dimethylformamide.

**Table 2:** ^1^H chemical shift assignments of PHB groups.

Structure	^1^H δ (ppm)
–O–CH(CH_3_)–CH_2_–CO–	δ_CH3_ 1.28, δ_CH2_ 2.48, δ_CH_ 5.27
–O–CH_2_–CH_2_–CH_2_–CO–	δα_CH2_ 1.70, δβ_CH2_ 1.42, δγ_CH2_ 3.40
–O–CH(C_2_H_5_)–CH_2_–CO–	δ_CH_ 5.13, δ_CH2_ 2.04, δ_CH3_ 0.85
HO–CH(CH_3_)–CH_2_–CO–	δ_CH3_ 1.20, δ_CH2_ 2.42, δ_CH_ 4.19
*trans*-CH_3_–CH=CH–CO–	δβ_CH_ 6.96, δα_CH_ 5.80, δ_CH3_ 1.87
*cis*-CH_3_–CH=CH–CO–	δβ_CH_ 6.33, δα_CH_ 5.75, δ_CH3_ 2.13
–O–CH(CH_3_)–CH_2_–CO–NH–CH_2_–CH_2_–NH_2_	δ_NH_ 4.03, δ_CH2NH_ 2.92, δ_CH3_ 1.21
–O–CH(CH_3_)–CH_2_–CO–NH–CH_2_–CH_2_–CH_2_–CH_2_–NH_2_	δ_NH_ 4.03, δ_CH2NH_ 3.0–2.75, δ_CH2CH2NH_ 1.60, δ_CH3_ 1.21
–O–CH(CH_3_)–CH_2_–CO–NH–CH_2_–CH_2_–OH	δ_OH_ 9.45, δ_NH_ 4.03, δ_CH2NH_ 1.66, δ_CH3_ 1.21

## Data Availability

The data presented in this study are openly available in Mendeley at http://dx.doi.org/10.17632/gt7txg9gzn (accessed on 13 December 2022).

## Supplementary Materials

The following supporting information can be downloaded at: https://www.mdpi.com/article/10.3390/polym14245481/s1, Figure S1: ^1^H NMR spectra; Figure S2: COSY correlation spectra.
