# Supplementary material for: Aminolysis of Poly-3-Hydroxybutyrate in N,N-Dimethylformamide and 1,4-Dioxane and Formation of Functionalized Oligomers
Source: Polymers (Basel). 2022 Dec 14;14(24):5481. doi: 10.3390/polym14245481 (PMC9780795; doi:10.3390/polym14245481)
Supplement: Supplementary file 1 [file polymers-14-05481-s001.zip › Figure S1.pdf]

(a)

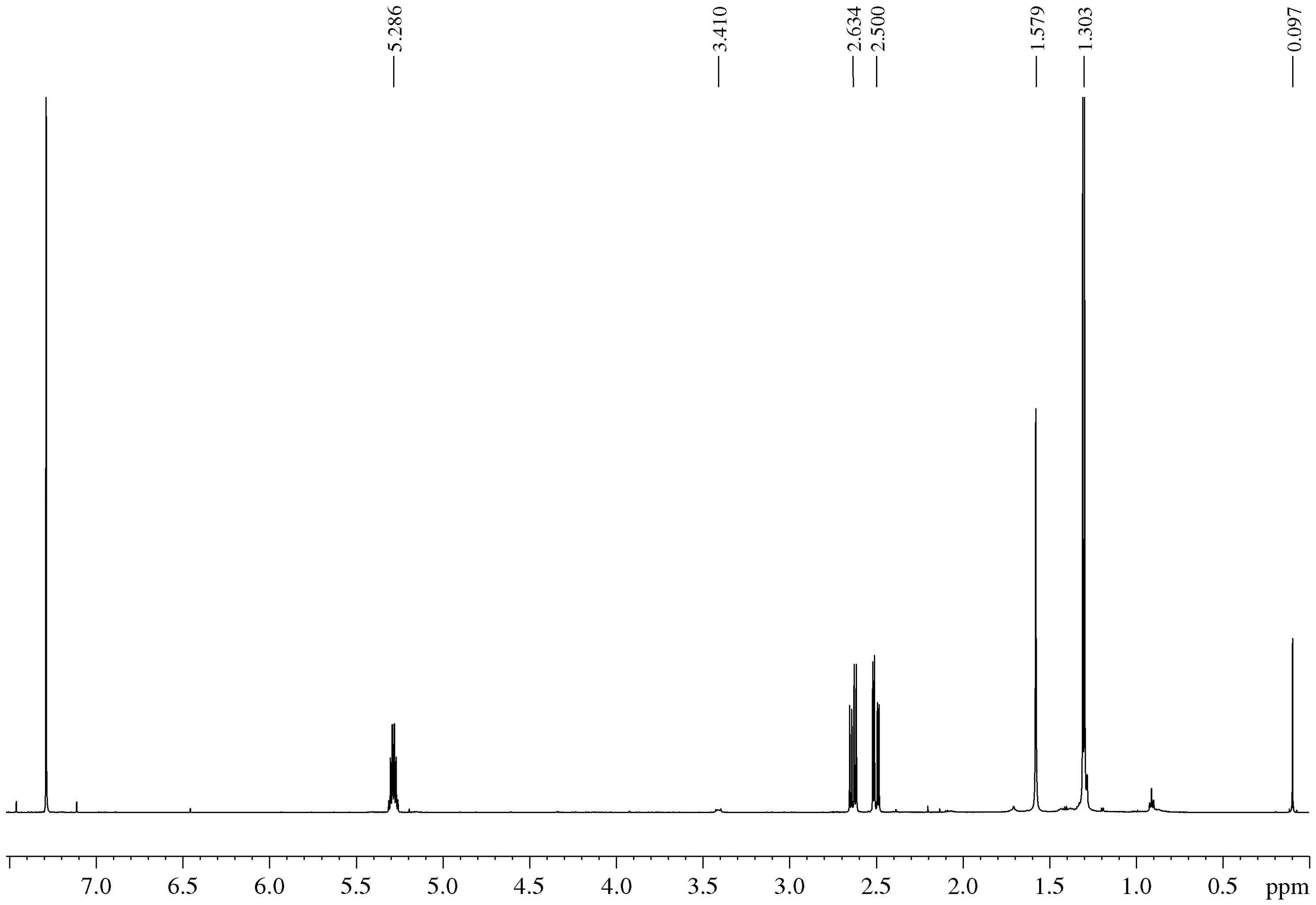

5.286
3.410
2.634
2.500
1.579
1.303
0.097
7.0
6.5
6.0
5.5
5.0
4.5
4.0
3.5
3.0
2.5
2.0
1.5
1.0
0.5
ppm

(b)

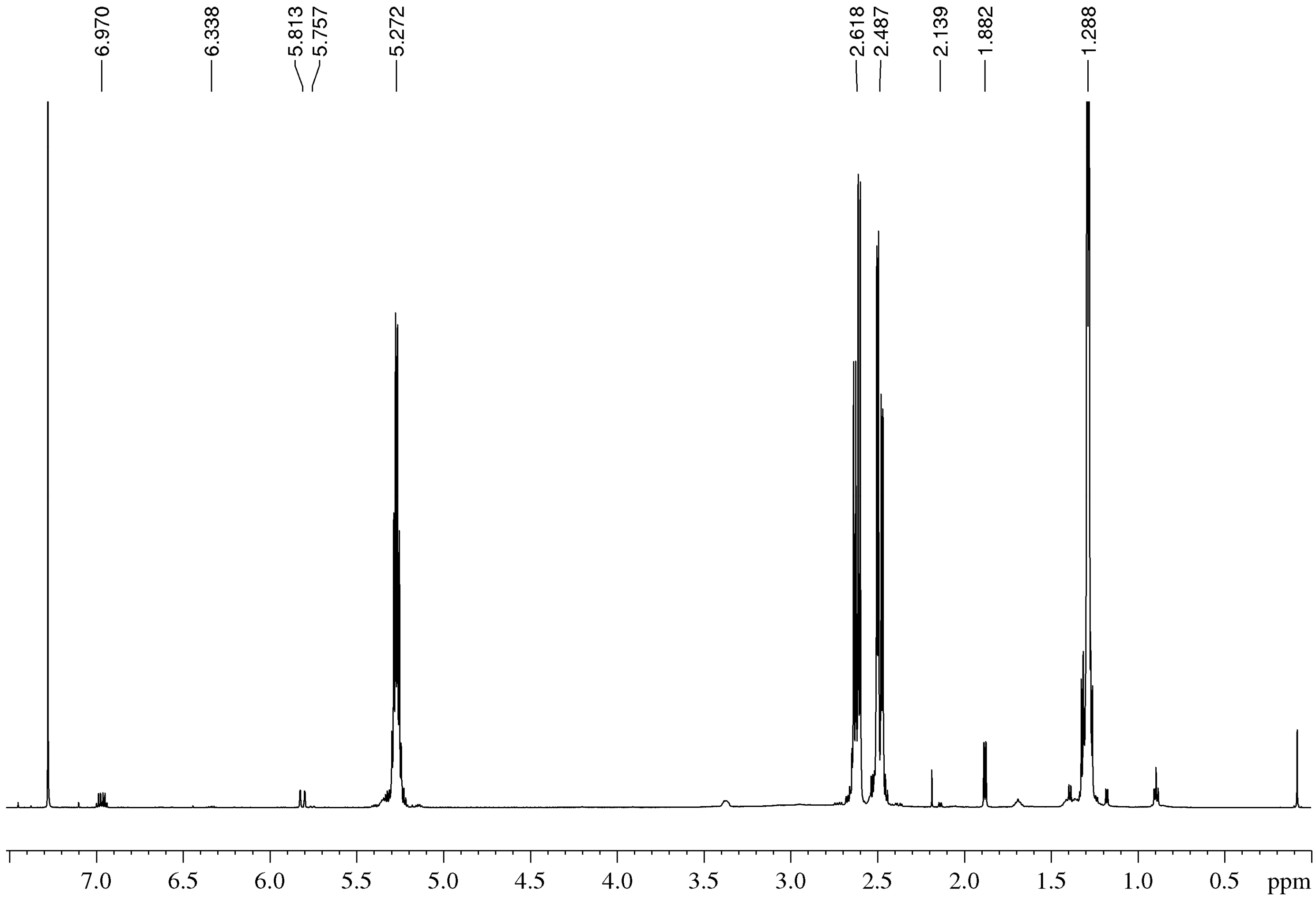
6.970
6.338
5.813
5.757
5.272
2.618
2.487
2.139
1.882
1.288
7.0
6.5
6.0
5.5
5.0
4.5
4.0
3.5
3.0
2.5
2.0
1.5
1.0
0.5
ppm

(c)

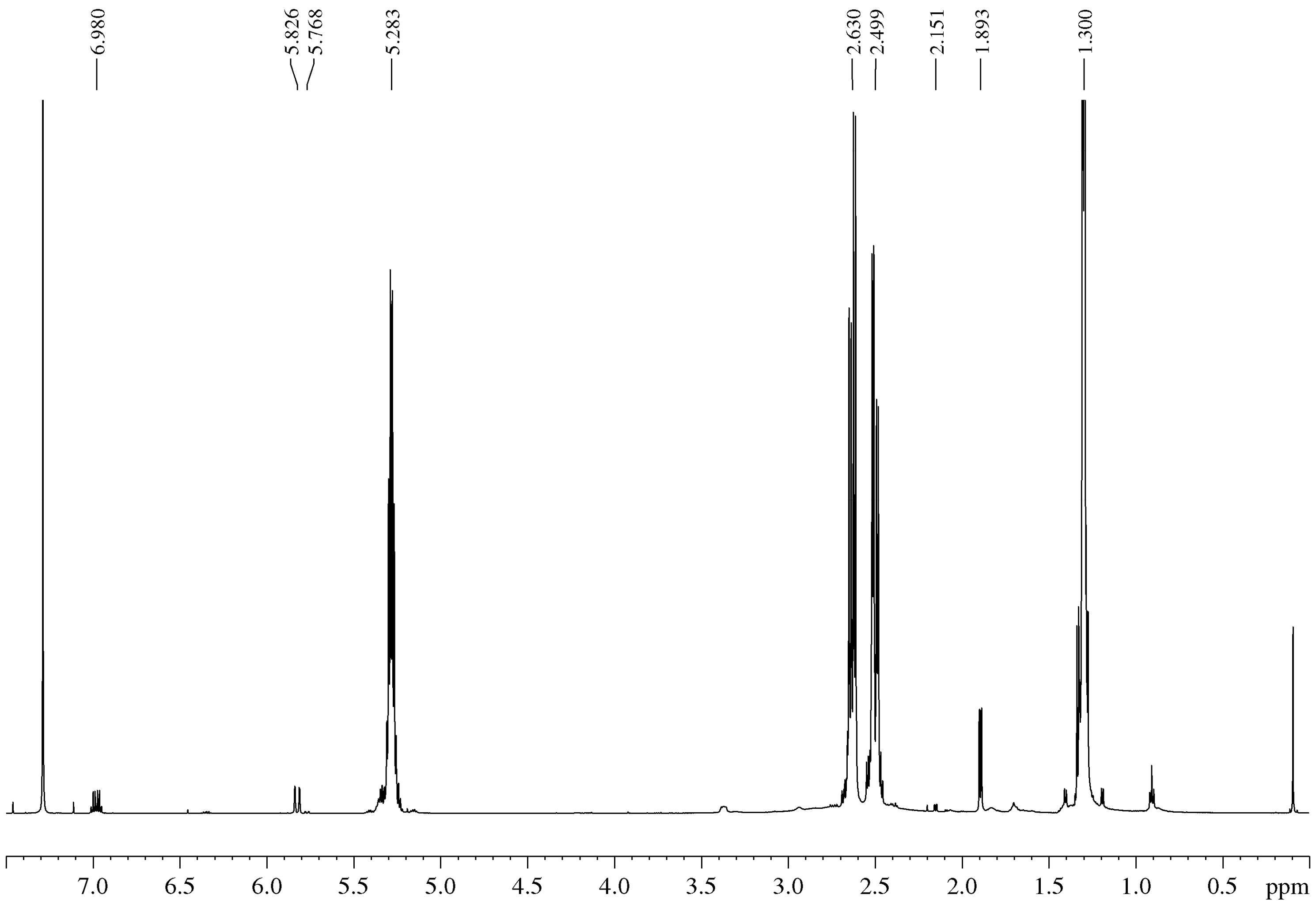
6.980
5.826
5.768
5.283
2.630
2.499
2.151
1.893
1.300
7.0
6.5
6.0
5.5
5.0
4.5
4.0
3.5
3.0
2.5
2.0
1.5
1.0
0.5
ppm

(**d**)

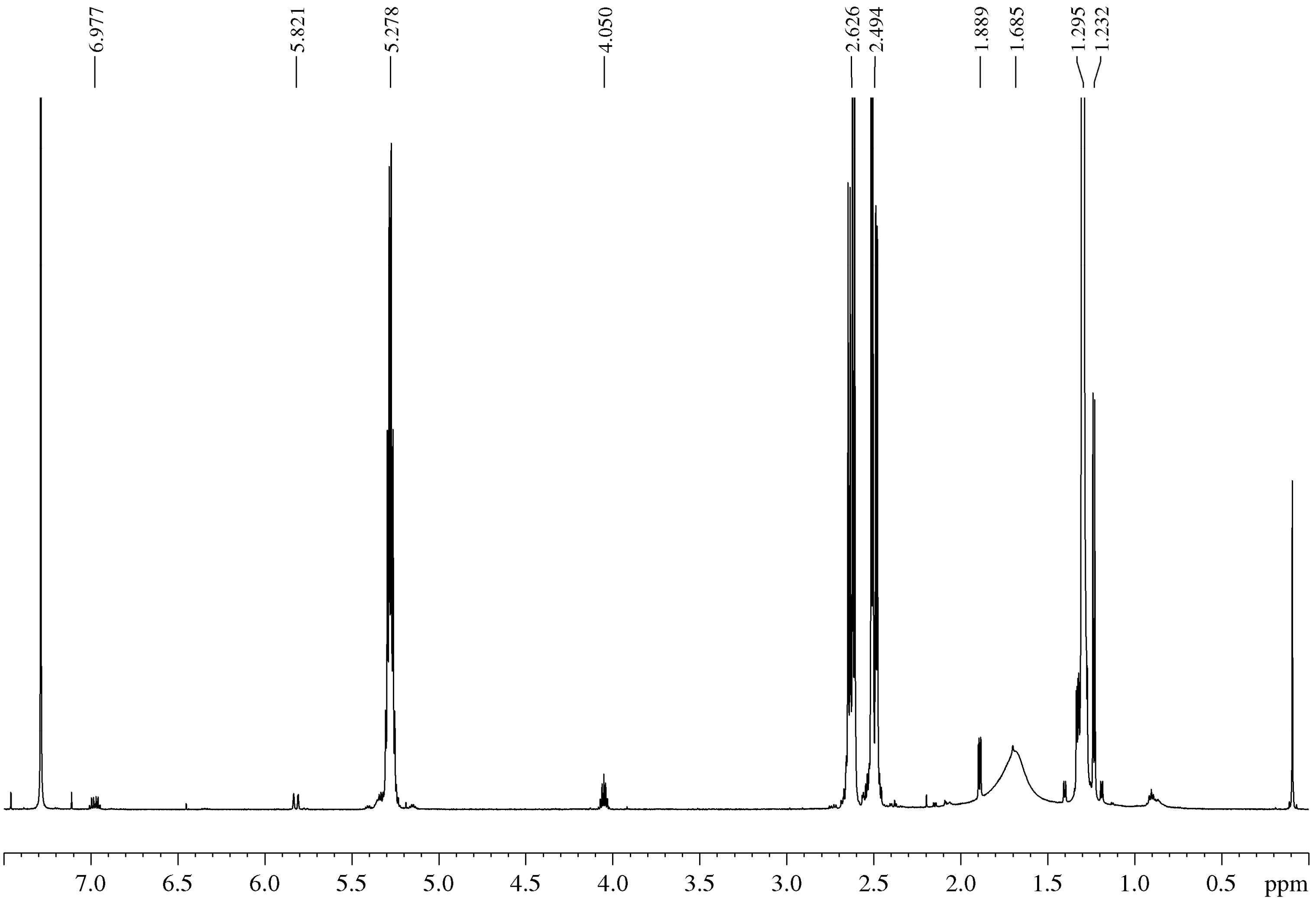
6.977
5.821
5.278
4.050
2.626
2.494
1.889
1.685
1.295
1.232
7.0
6.5
6.0
5.5
5.0
4.5
4.0
3.5
3.0
2.5
2.0
1.5
1.0
0.5
ppm

(e)

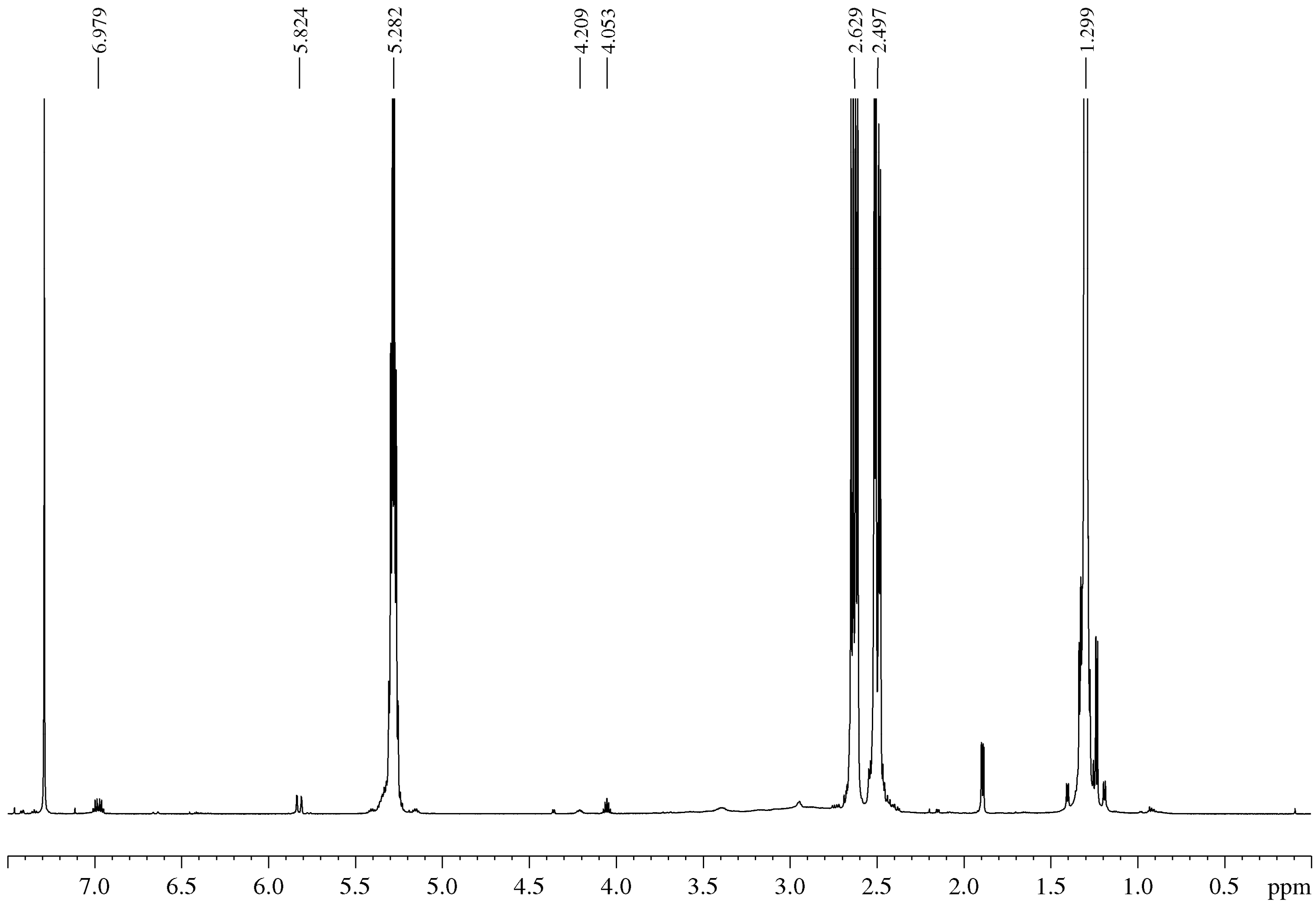
6.979
5.824
5.282
4.209
4.053
2.629
2.497
1.299
7.0
6.5
6.0
5.5
5.0
4.5
4.0
3.5
3.0
2.5
2.0
1.5
1.0
0.5
ppm

(f)

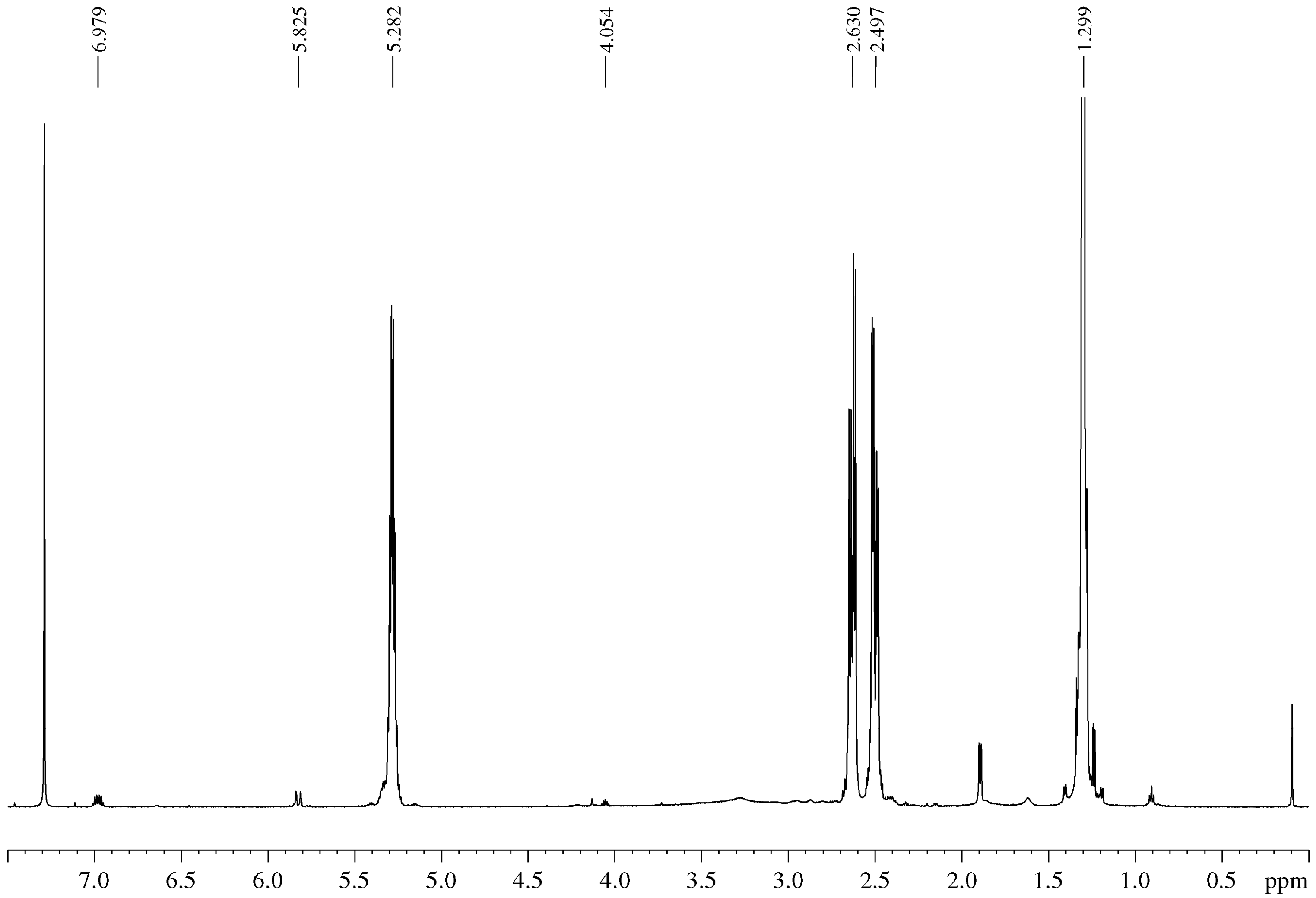
6.979
5.825
5.282
4.054
2.630
2.497
1.299
7.0
6.5
6.0
5.5
5.0
4.5
4.0
3.5
3.0
2.5
2.0
1.5
1.0
0.5
ppm

(g)

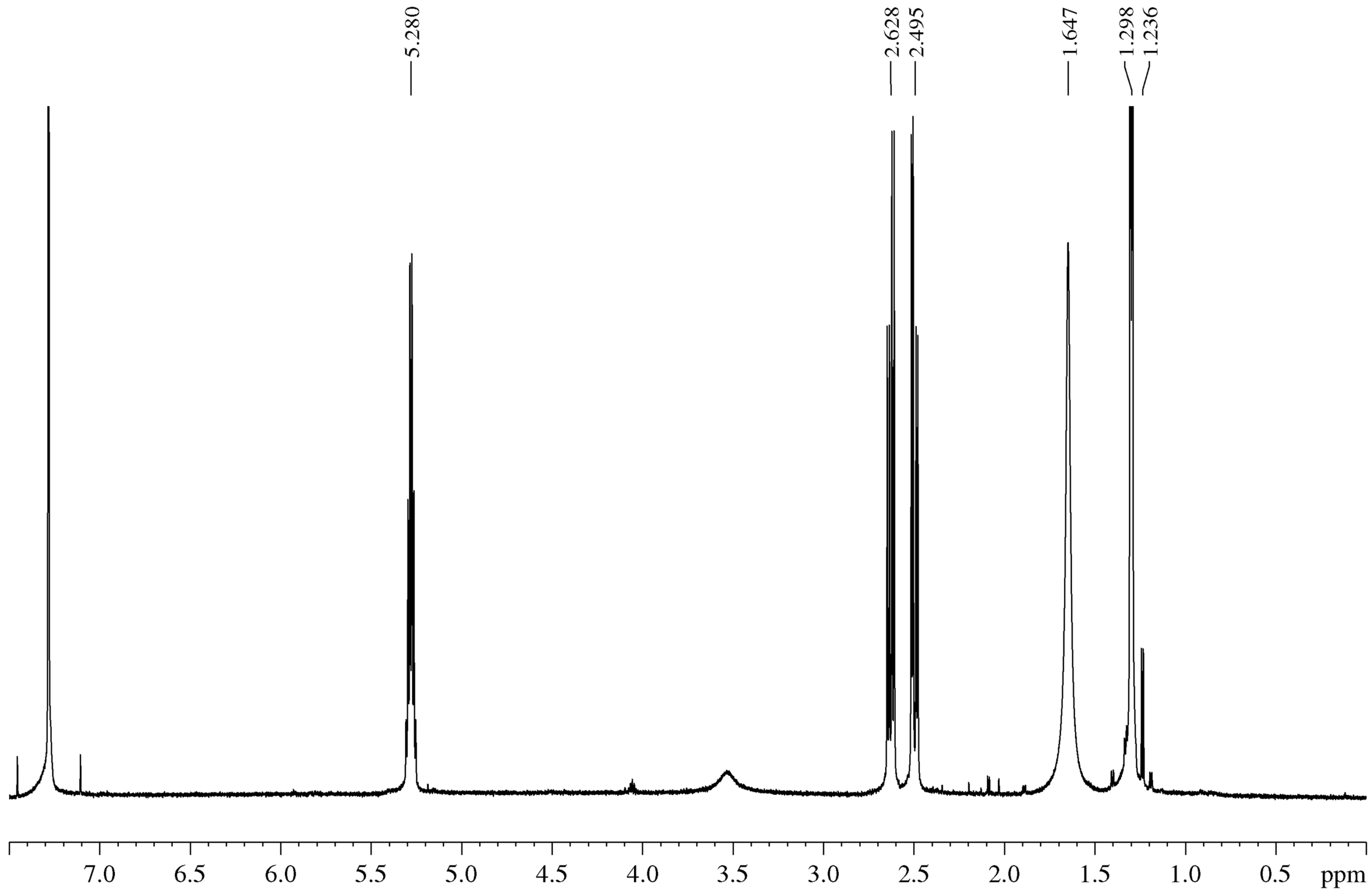
5.280
2.628
2.495
1.647
1.298
1.236
7.0
6.5
6.0
5.5
5.0
4.5
4.0
3.5
3.0
2.5
2.0
1.5
1.0
0.5
ppm

Figure S1. ^{1}H NMR spectra: (**a**) – initial PHB; oligomers after treatment: (**b**) – EDA/DMF; (**c**) – DAB/DMF; (**d**) – MEA/DMF; (**e**) – EDA/dioxane; (**f**) – DAB/dioxane; (**g**) – MEA/dioxane.
