# Supplementary material for: Aminolysis of Poly-3-Hydroxybutyrate in N,N-Dimethylformamide and 1,4-Dioxane and Formation of Functionalized Oligomers
Source: Polymers (Basel). 2022 Dec 14;14(24):5481. doi: 10.3390/polym14245481 (PMC9780795; doi:10.3390/polym14245481)
Supplement: Supplementary file 1 [file polymers-14-05481-s001.zip › Figure S2.pdf]

(a)

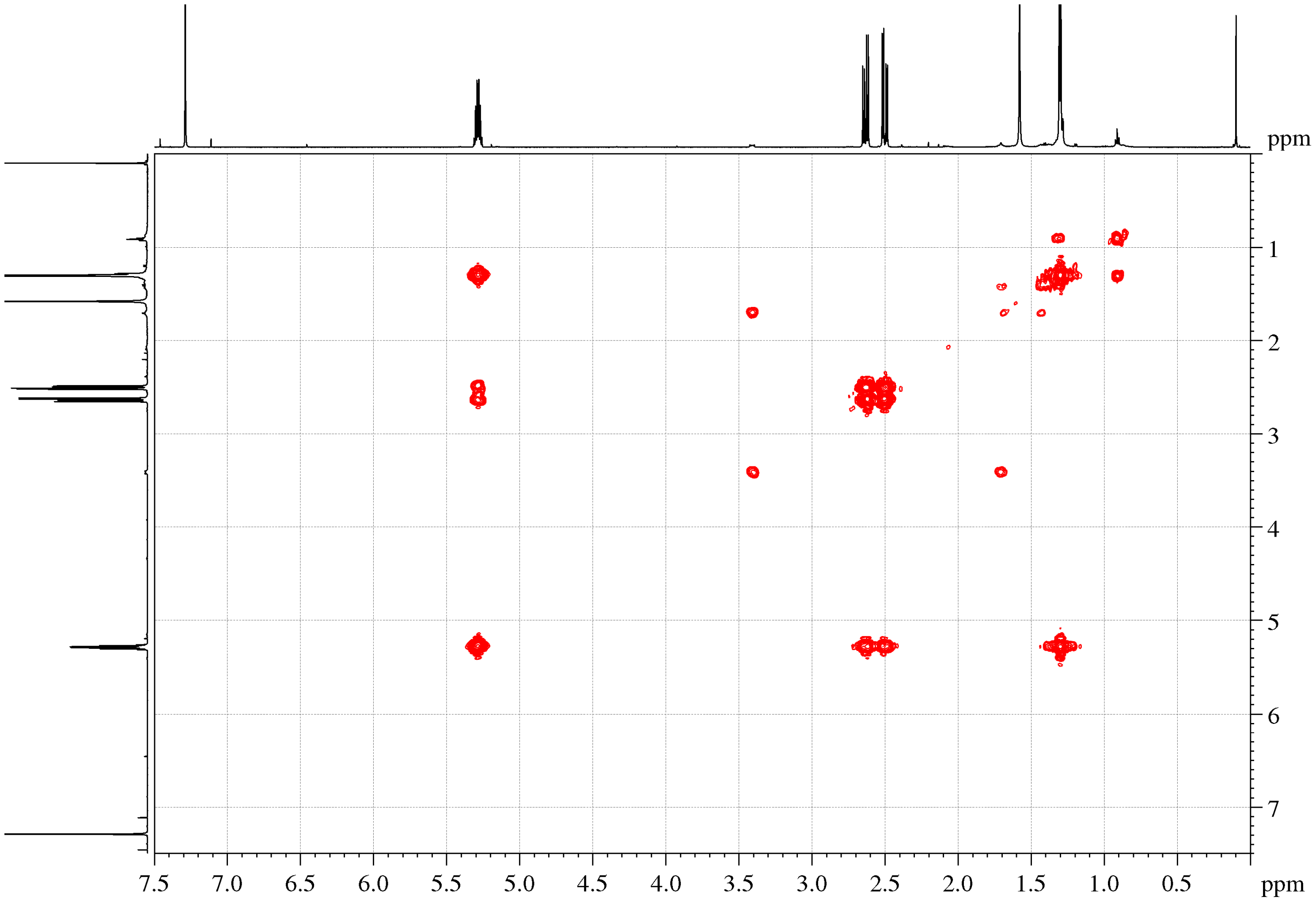

(b)

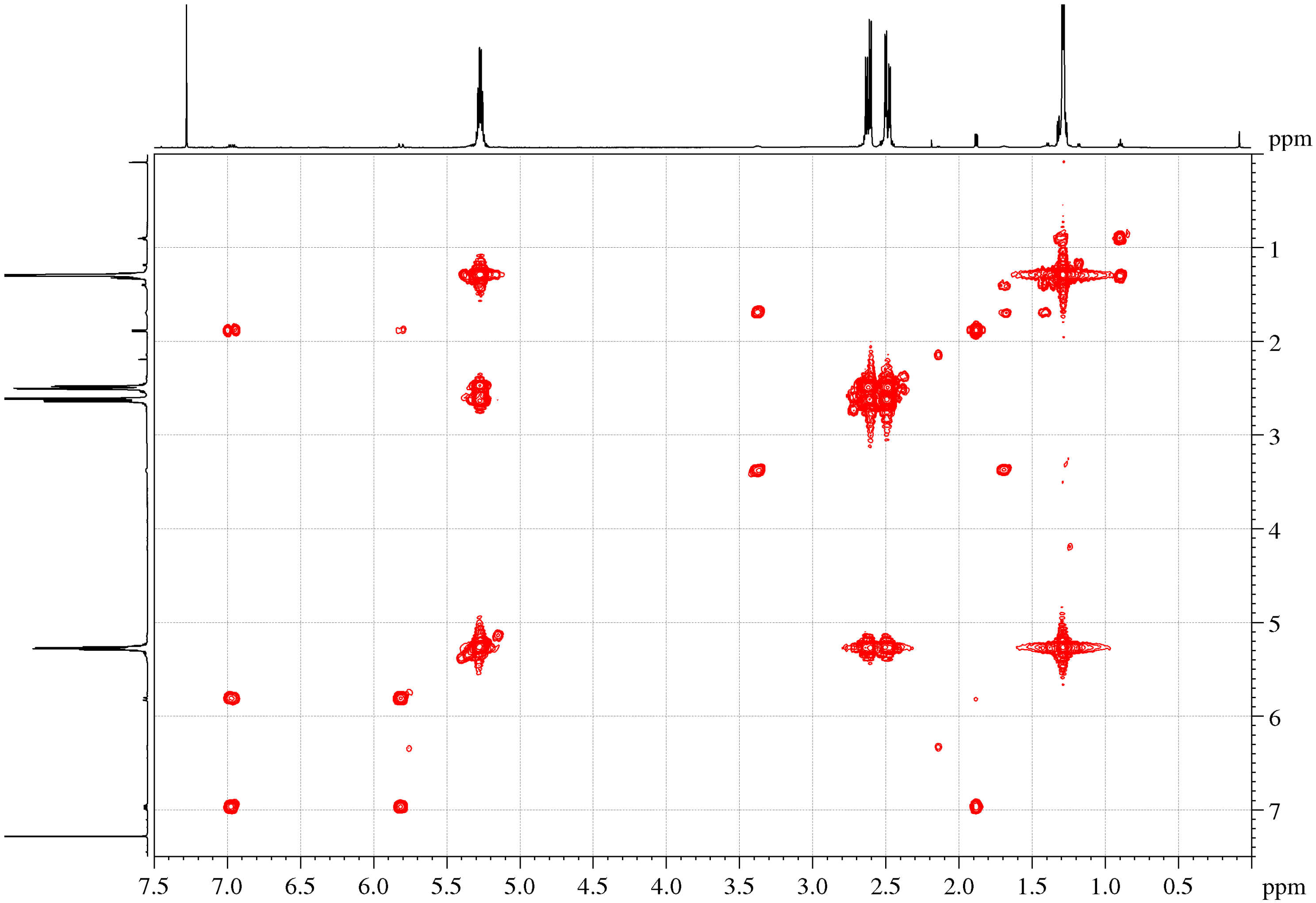

(c)

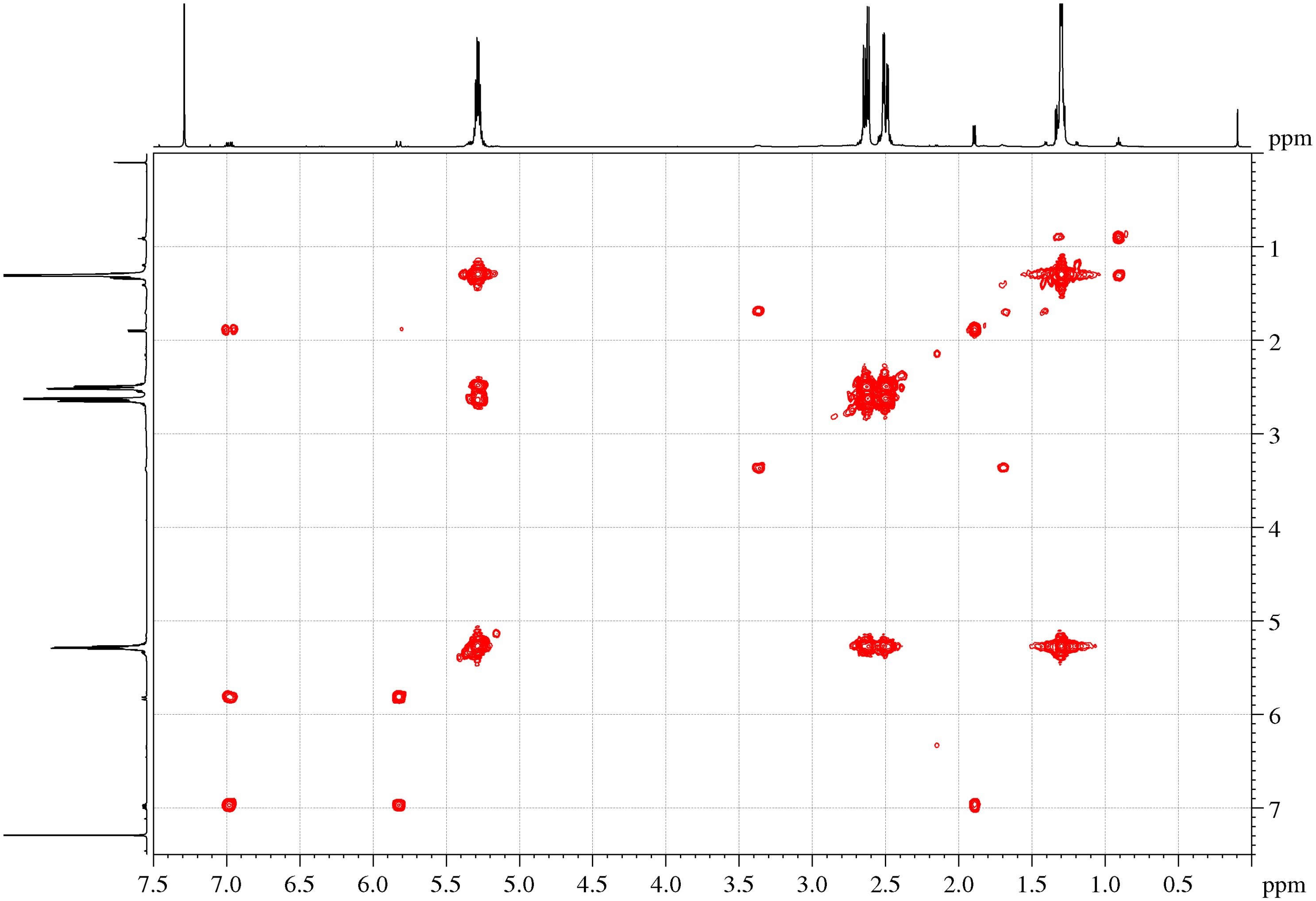

(d)

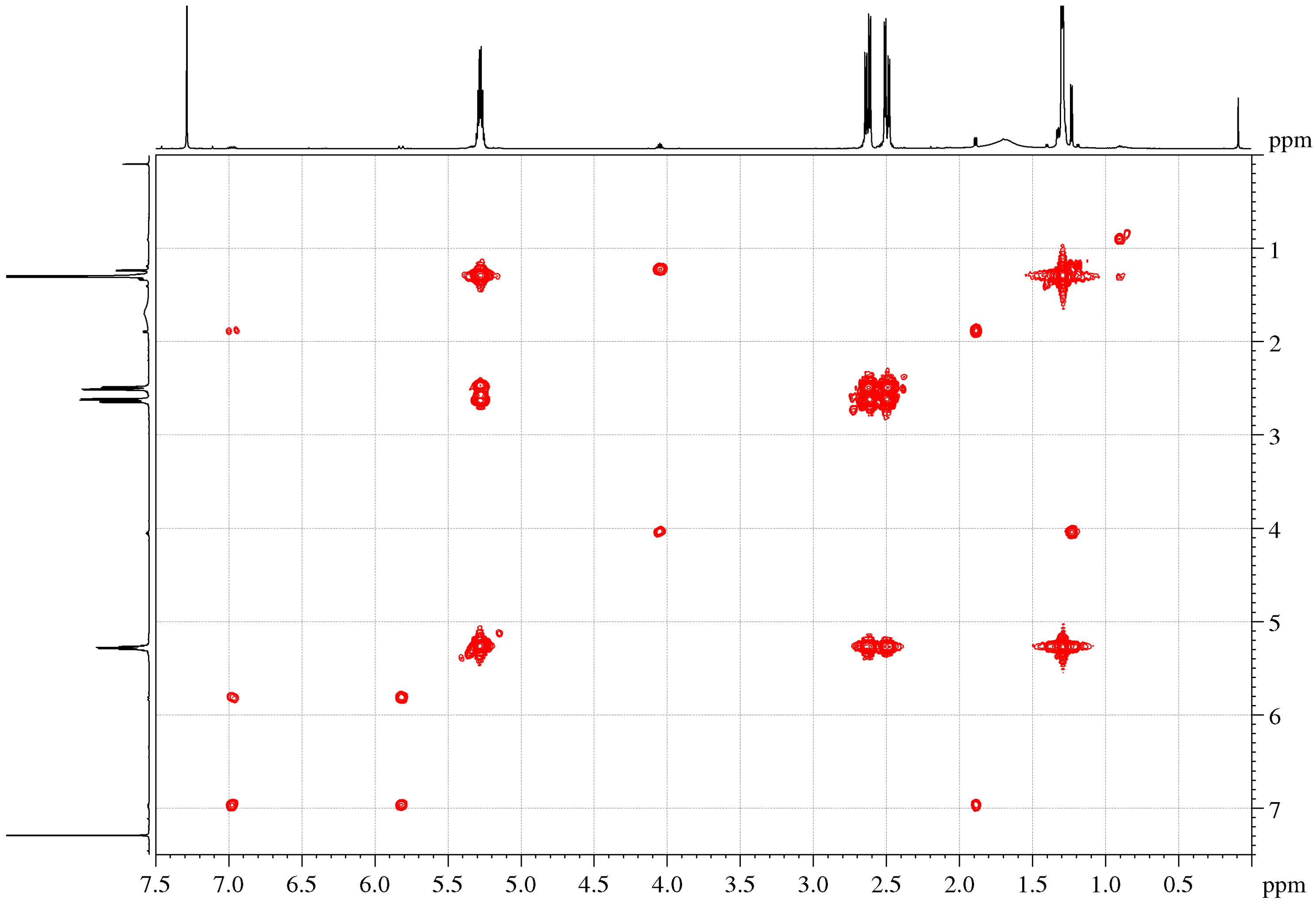

(e)

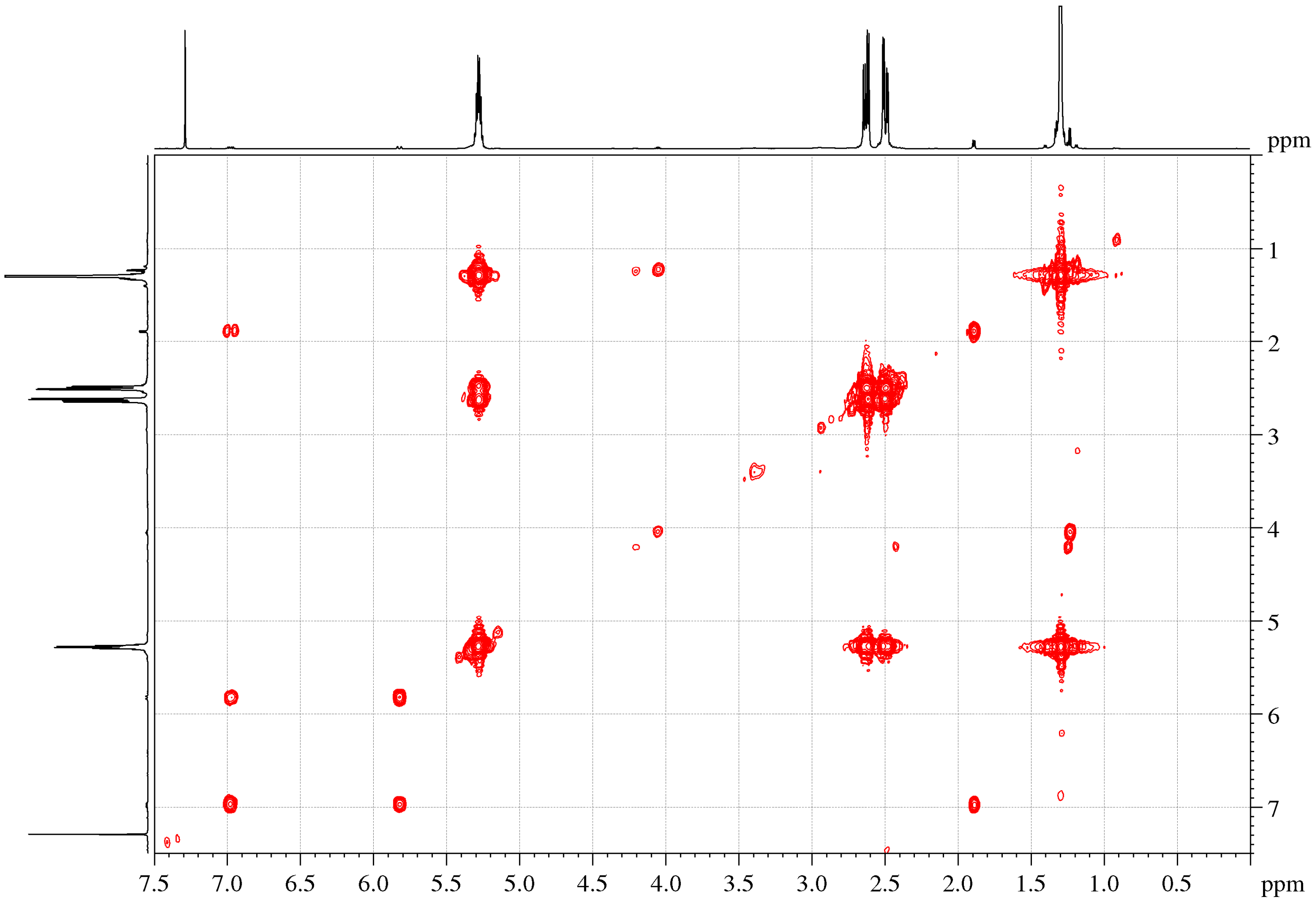

(f)

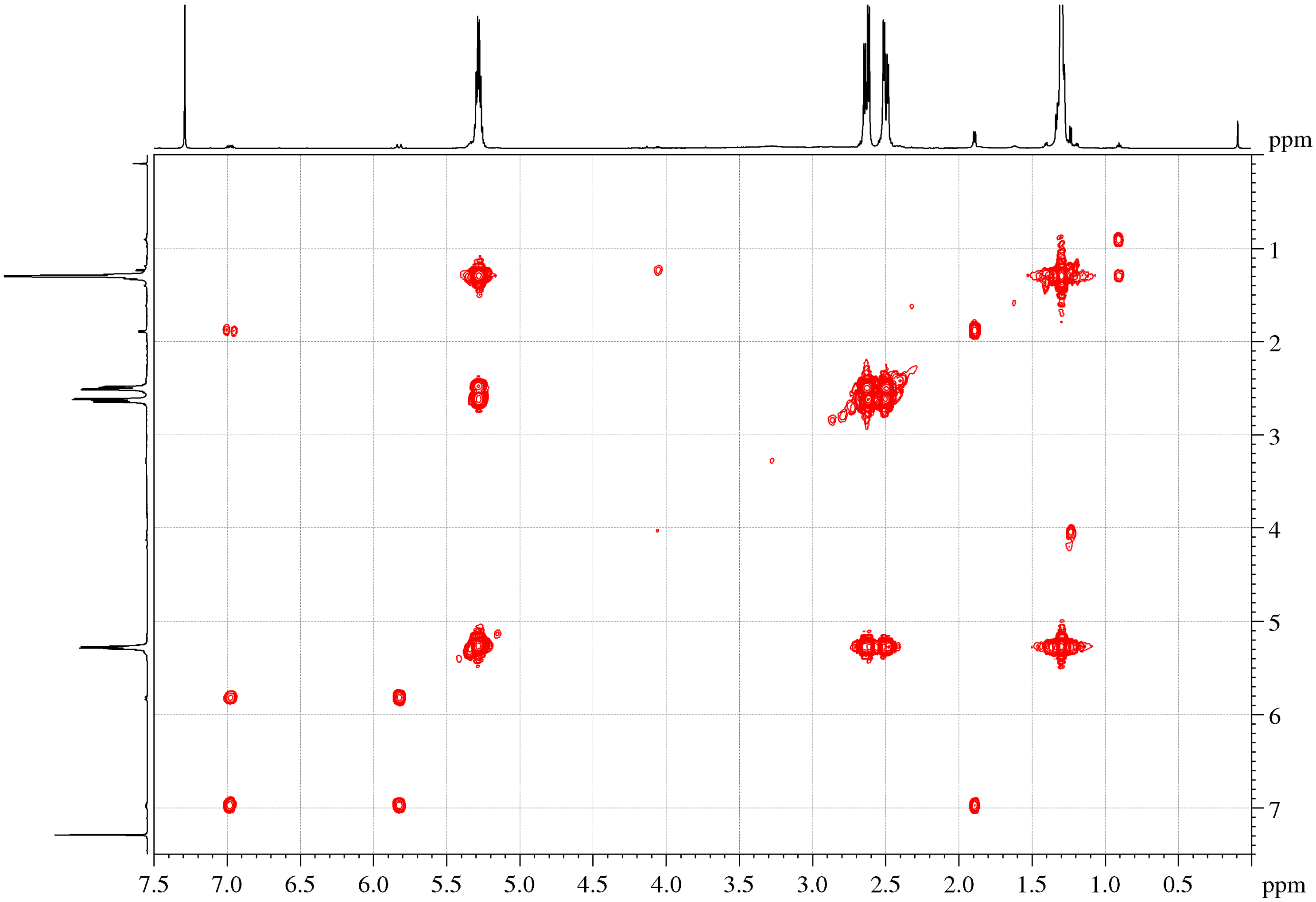

(g)

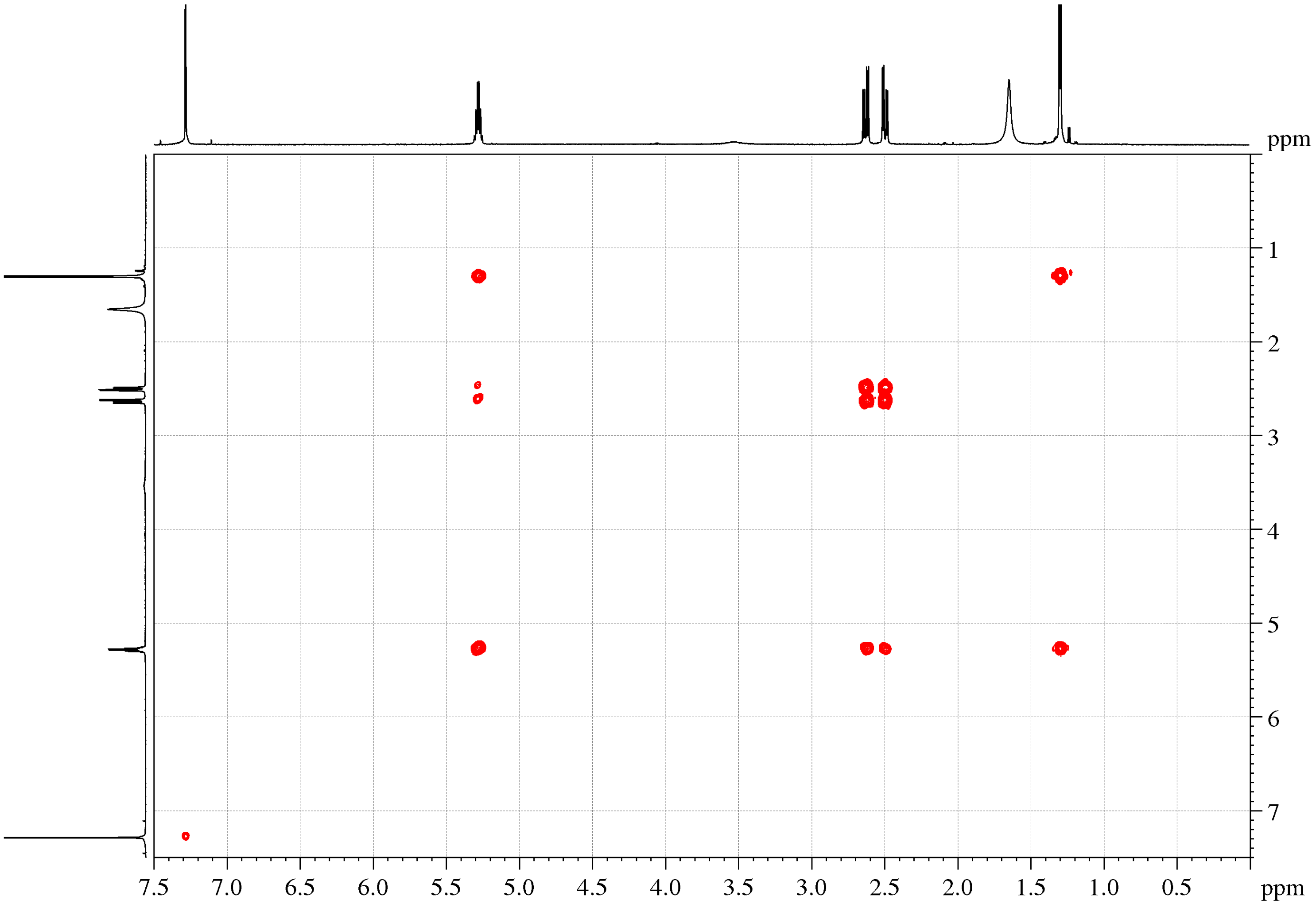

Figure S1. COSY correlation spectra: (**a**) – initial PHB; oligomers after treatment: (**b**) – EDA/DMF; (**c**) – DAB/DMF; (**d**) – MEA/DMF; (**e**) – EDA/dioxane; (**f**) – DAB/dioxane; (**g**) – MEA/dioxane.
